# Affections of the Gum in Relation to Other Diseases

**Published:** 1885-02

**Authors:** 


Affections of the Gum in Relation to Other Diseases.—Dr. Kaczorowski (*Przeglad Lekarski*, Nos. 28 and 29, 1884, and *Vratch*, No. 32, 1884) draws attention to a connection existing between gingival affections and certain other diseases. In four of his cases, chronic gingivitis caused the occurrence of hallucinations, melancholia, nervous excitement, and insanity. Extraction of destroyed teeth and appropriate treatment of the inflamed foul gums were followed, in each of the cases, by restoration of health of the nervous system. Further, the author saw several instances where affection of the gum led to general septicæmia. He thinks generally that premature senile debility of the organism may often depend upon dental caries, leading to absorption into the system of septic products of slow decomposition.—*London Medical Record.*

